# Effects of a single mental chronometry training session in subacute stroke patients – a randomized controlled trial

**DOI:** 10.1186/s13102-020-00212-w

**Published:** 2020-10-22

**Authors:** Joachim Liepert, Jana Stürner, Imke Büsching, Aida Sehle, Mircea A. Schoenfeld

**Affiliations:** 1 Department of Neurorehabilitation, Kliniken Schmieder, Zum Tafelholz 8, 78476 Allensbach, Germany; 2 Rehaklinik Bellikon, Bellikon, Switzerland; 3 Department of Neurorehabilitation, Kliniken Schmieder, Heidelberg, Germany; 4 Department of Experimental Neurology, Medical Faculty, Otto-von-Guericke University Magdeburg, Magdeburg, Germany; 5 Department of Behavioral Neurology, Leibniz Institute for Neurobiology, Magdeburg, Germany

**Keywords:** Motor imagery, Mental chronometry, Stroke, Motor execution, Motor excitability

## Abstract

**Background:**

Motor imagery training might be helpful in stroke rehabilitation. This study explored if a single session of motor imagery (MI) training induces performance changes in mental chronometry (MC), motor execution, or changes of motor excitability.

**Methods:**

Subacute stroke patients (*n* = 33) participated in two training sessions. The order was randomized. One training consisted of a mental chronometry task, the other training was a hand identification task, each lasting 30 min. Before and after the training session, the Box and Block Test (BBT) was fully executed and also performed as a mental version which served as a measure of MC. A subgroup analysis based on the presence of sensory deficits was performed. Patients were allocated to three groups (no sensory deficits, moderate sensory deficits, severe sensory deficits). Motor excitability was measured by transcranial magnetic stimulation (TMS) pre and post training. Amplitudes of motor evoked potentials at rest and during pre-innervation as well as the duration of cortical silent period were measured in the affected and the non-affected hand.

**Results:**

Pre-post differences of MC showed an improved MC after the MI training, whereas MC was worse after the hand identification training. Motor execution of the BBT was significantly improved after mental chronometry training but not after hand identification task training. Patients with severe sensory deficits performed significantly inferior in BBT execution and MC abilities prior to the training session compared to patients without sensory deficits or with moderate sensory deficits. However, pre-post differences of MC were similar in the 3 groups. TMS results were not different between pre and post training but showed significant differences between affected and unaffected side.

**Conclusion:**

Even a single training session can modulate MC abilities and BBT motor execution in a task-specific way. Severe sensory deficits are associated with poorer motor performance and poorer MC ability, but do not have a negative impact on training-associated changes of mental chronometry. Studies with longer treatment periods should explore if the observed changes can further be expanded.

**Trial registration:**

DRKS, DRKS00020355, registered March 9th, 2020, retrospectively registered

## Background

Motor imagery (MI) is a process that involves the mental execution of an action in the absence of movement, and it activates neural structures and processes similar to those activated when certain movements are actually performed [1, 2]. Different aspects contribute to MI abilities. The subject needs to be able to imagine actions in a three-dimensional space [3]. He or she should also be able to correctly estimate the duration of an imagined movement. This ability addressing the temporal structure of simulated actions is called mental chronometry (MC). MC involves the comparison of movement times during imagined and executed motor tasks [3]. It has been shown to be a reliable tool for screening for MI abilities [4]. A third aspect of MI is the vividness of motor imagery. This ability is typically addressed by self-assessment questionnaires (e.g., [5–7]).

Mental practice (MP) is a training method using MI elements and has been administered to stroke patients as an adjunct therapeutic approach for more than 15 years (e.g., [8, 9]). Current evidence suggests that most [3, 10–12] but not all stroke patients [13–16] have MI abilities similar to healthy subjects. However, controlled treatment trials have shown conflicting results. Recent reviews concluded that a trend in support of MP as a treatment option for upper extremity rehabilitation was evident [17] but that heterogeneity in methodological quality was too high to recommend MP in general [18].

The primary goal of this study was to investigate if even a single session of MI training produces detectable changes in MC performance, motor performance and motor excitability. In particular, we explored if changes in performance and/or excitability are task-specific. As secondary goal, we explored if the effects of a single MI training are influenced by impairments of sensory functions.

## Methods

The same group of stroke patients participated in a MI training and, as a control condition, in a hand identification task training that addresses imaging (mental rotation) in three-dimensional space. Transcranial magnetic stimulation (TMS) techniques were used to assess changes of motor excitability. It has been shown that, during a motor imagery task, motor excitability increases specifically for those muscles involved in the task [19], Excitability changes are typically assessed by measuring the amplitudes of motor evoked potentials. In a preceding study we demonstrated that stroke patients with a severe sensory deficit had less motor excitability increases during a motor imagery task than stroke patients with a pure motor deficit [20]. In the present study, we did not examine motor excitability during but before and after the training period.

### Patients

Altogether, 33 stroke patients (13 female, mean age: 63 ± 11 years, 16 with right-sided paresis, time since onset: 2.1 ± 1.1 months) were included after having given consent to participate in the study. Participants were recruited during inpatient neurological rehabilitation at Kliniken Schmieder, Allensbach, Germany. They stayed in the hospital for at least 4 weeks and had 3 treatment hours per day, 5 days a week. Treatments focused on hand and arm function for approximately 2 h per day and were similar for all patients regarding the amount and duration of therapy. For this trial, the patients in addition participated in a motor imagery training and a hand identification training. Both kinds of trainings have been used in stroke patients before [10, 15, 20, 21].

Our inclusion criteria were the ability to understand the instructions, the willingness to participate, an ischemic or hemorrhagic stroke < 6 months ago as proven by cranial computed tomography or magnetic resonance imaging of the brain and the ability to grasp and release blocks that are typically used in a classical Box and Block Test [22]. The cubes have an edge length of 2.5 cm. Subjects should be able to grasp and release at least 15 cubes. There were no restraints regarding time for accomplishing the task or the way of grasping.

Exclusion criteria were hemianopia, spatial neglect, anosognosia, severe other illness that could interfere with the ability to participate actively. Exclusion criteria for TMS application were a history of epileptic seizures, pregnancy, metallic implants in the brain and heart pace makers.

The location of the lesion was determined based on either magnetic resonance imaging or computer tomography. Lesions were stratified as being “subcortical” without any evidence of an involvement of cortical areas or as being “cortical/subcortical” with an involvement of cortical and subcortical areas.

Patient recruitments were started in March 2017 and, after reaching the estimated number of participants, terminated in July 2018.

### Measures

#### Somatosensory testing

The sensory testing included detection of light touch, localization of touch and assessment of vibration sense as described earlier [15]. Detection of light touch was assessed with a cotton carrier at the fingertip of Digit II of the affected hand without visual control by the patient. It was noted if the patient felt the light touch on the affected hand immediately, only when applying pressure, or if the carrier couldn’t be felt at all. To assess localization of touch, three different fingers of the affected hand were touched, again without visual control by the patient. Distinction was made if the patient detected the touch at all fingers correctly, if there was a mistake in one or two fingers, or no localization of touch was possible at all. A tuning fork with a scale of 0 to 8 served to test the vibration sense on the dorsal side of metacarpal joint of Digit II of the affected hand. 0 to 3 was defined as severe sensory loss, 4–6 as moderate sensory loss, and 7–8 as no sensory deficit in vibration sense.

#### Box and block test (BBT)

A modified version of the BBT with 15 blocks was used for evaluation of motor performance and MC ability. The BBT is a widely used means of measuring upper extremity dexterity, including grasping, moving an object over a barrier and releasing it [22]. This test is frequently used as a measure of dexterity and has been shown to be valid and reliable [23–25]. It has been used to measure motor imagery abilities in preceding studies [20, 26]. The task requires the ability to grasp and release objects and to perform shoulder and elbow movements because the patient has to raise his arm in order to surmount the partition of the box with a height of 15 cm.

The patients first perform the BBT mentally and then execute it as a motor task with one hand. For mental performance of the BBT, the patient received an auditory go-signal from the examiner and indicated orally when he or she had completed the task. Motor imagery (MI) and motor execution (ME) of the BBT were first performed for the affected hand and then for the non-affected one. The sequence of motor imagery and affected side first was chosen in order to avoid any influence and learning experience from motor execution and from the unaffected side. The times the patient needed to perform the task mentally and physically were measured by a stopwatch. MC ability was calculated as a ratio of ([motor execution time– motor imagery time]/ motor execution time) [15]. The reason for applying this ratio was to account for the influence of different ME times. For example, a difference of 3 s between MC and ME indicates a worse MC ability if ME takes 15 s than when it takes 30 s. In dependence on whether ME or MI time was shorter, the ratio could either be a positive or a negative value. Since we were exclusively interested in the absolute difference, all ratios were expressed as positive values. The closer to zero a ratio is, the higher is the congruency between MI and ME. In the next step, pre-training ratios were subtracted from post-training ratios. In this case, the change of ratio was relevant: negative values indicated that post-training values had been closer to zero than pre-training ratios. Thus, negative values indicate an improved MC performance post-training whereas positive values show a deterioration in MC performance.

BBT measures were done before and after both training sessions.

#### Transcranial magnetic stimulation (TMS)

TMS was performed with a circular coil. Recordings were taken from the first dorsal interosseous muscle (FDI) on both hands. Both hemispheres were studied consecutively. First, the optimal coil position, defined as the place where motor evoked potentials (MEPs) could be evoked with the lowest stimulus intensity, was determined. This coil position was marked with ink on the skin to ensure an exact repositioning of the coil throughout the experiment. Then, the motor threshold was identified. Resting motor threshold (MT) was defined as the stimulus intensity needed to produce MEPs with a size of 50–100 μV in 5 out of 10 consecutive trials during complete muscle relaxation [27].

Transcranial magnetic stimulus intensity was set at 130% of the individual motor threshold. Six motor evoked potentials were recorded with the target muscle at rest. Another six MEPs as well as the cortical silent period (cSP) were collected during pre-innervation of the target muscle with approximately 20% of the maximum voluntary contraction force.

Recordings were stored (Viking IV, Viasys Comp.) and analysed off-line. MEPs were measured peak-to-peak, and a mean amplitude was calculated. The cSP duration was defined from the beginning of the MEP until the re-occurrence of EMG activity with an amplitude of at least 30% of the pre-stimulation EMG activity.

TMS was done before and after both training sessions.

The persons performing the measurements were blinded regarding the type of training.

The primary outcome measure was the change of MC ability, secondary outcomes measures were changes of ME abilities and changes of TMS parameters.

### Training

#### Motor imagery training (MI)

The BBT was used in the same way as described above. Patients were advised to exclusively imagine performing the task with the affected upper limb. A total of 15 blocks was placed in the box, arranged in 5 rows of 3 blocks. The patient received an auditory go-signal from the examiner and indicated orally when he or she had completed the task. The time between go-signal and stop-signal was measured with a stop-watch and recorded. This procedure was repeated as often as possible until 30 min were over. The patient did not receive any information about his or her level of performance. We chose to give no immediate feedback in order to imitate the typical clinical setting when MI is used as an add-on therapy.

#### Hand identification task training (HIT)

In this mental rotation task, the subject watches a hand that is displayed in 8 different orientations on a PC screen and has to decide whether it is a right or a left hand by pressing the appropriate button on the keypad. Altogether, 40 hand images per run were presented, with four runs being performed. The results were analyzed with respect to the correctness of the answer and the time interval between target presentation and button press (response time). Patients used their unaffected hand to press the button.

Both trainings were performed for 30 min. The sequence of trainings (first MI training followed by HIT training or vice versa) was randomized for each patient. Randomization was done by a person otherwise not involved in the study. A closed envelope that contained the information about the sequence for each single patient was given to the persons that performed the training sessions.

A minimum of 3 days was chosen as the interval between the sessions.

#### Subgroups according to the degree of sensory impairment

Based on sensory assessments the patients were subdivided into three groups. Group 1 patients did not have any sensory deficits. Group 2 comprised patients with mild to moderate sensory loss while group 3 was formed by the patients with severe sensory loss.

### Statistical analysis

Statistical calculations were performed with IBM SPSS, version 25. A Shapiro Wilk test was performed to analyze if the data show a normal distribution. Only the ratios (ME-MI)/ME showed a normal distribution. BBT results and the TMS parameters were not normally distributed. For analysis of the non-normally distributed data Friedman tests were performed. Affected and non-affected side were calculated separately. The ratios were analyzed with a paired sample t test. For analysis of the 3 patient groups with different degrees of sensory deficits an analysis of variance (ANOVA) was calculated for the normally distributed MC data. The non-normally distributed BBT motor execution data were analyzed with Kruskal-Wallis test and, as posthoc analysis, with Mann-Whitney tests.

The level of statistical significance was assumed with *p* < 0.05.

## Results

Clinically, 10 patients presented a pure motor disturbance, 18 patients had a moderate impairment of sensory functions and 5 patients suffered from severe sensory deficits. Radiological results indicated subcortical hemorrhage in 8 patients and ischemic strokes in 25 patients. Altogether, 22 patients had pure subcortical lesions, the other 11 patients showed an additional involvement of cortical areas.

### Mental chronometry ratios

Calculation of pre-post differences of MC ratios showed that only the group that had performed the MC training had a smaller MC ratio after the intervention whereas MC ratios increased after HIT training (MI training: − 0.05 ± 0.16; HIT: 0.05 ± 0.15). This difference was significant (*p* = 0.015).

### BBT motor execution

On the paretic side, a significant difference between pre and post values was found for MI training but not for HIT training (Table 1). However, post values did not differ significantly between both exercises (*p* = 0.699). No significant differences between pre and post values were found on the unaffected side.

**Table 1:** Motor execution times, measured in seconds, before (pre) and after (post) a single session of mental chronometry training (MC) and hand identification training (HIT). Mean values ± standard deviations are presented. n.s., not significant

	Box and block test			
	MC group		HIT group	
	Affected	Unaffected	Affected	Unaffected
Pre	27.4 ± 12.7	12.5 ± 2.1	27.3 ± 14.4	12.5 ± 2.3
post	25.5 ± 12.7	12.5 ± 2.6	26.8 ± 14.6	12.3 ± 2.4
*P* value (pre – post)	0.006	0.67 (n.s.)	0.199 (n.s.)	0.41 (n.s.)

### Sensory deficits

The three subgroups of patients (no sensory deficit [Group 1], moderate deficit [Group 2], severe deficit [Group 3]) were analyzed in relation to BBT motor execution, MC abilities prior to the training sessions and MC pre-post differences.

The three groups differed significantly in their motor performance (Group 1; 19.1 ± 3.8 s; Group 2: 26.0 ± 11.8 s, Group 3: 45.8 ± 14.6 s; *p* < 0.001. Group 1 versus Group 2: *p* = 0.102; Group 1 versus Group 3: p < 0.001; Group 2 versus Group 3: p < 0.001) and in their MC abilities prior to HIT training and MI training (Group 1: 0.27 ± 0.17; Group 2: 0.26 ± 0.17; Group 3: 0.41 ± 0.15; F = 3.788; *p* = 0.028. Group 1 versus Group 3: *p* = 0.078; Group 2 versus Group 3: *p* = 0.03; Group 1 versus Group 2: not significant) but were not significantly different in the mental chronometry pre-post differences (Group 1: MI training: − 0.049 ± 0.1; HIT: 0.019 ± 0.19; Group 2: MI training: − 0.054 ± 0.17; HIT: 0.066 ± 0.13; Group 3: MI training: − 0.062 ± 0.2; HIT: 0.04 ± 0.11).

### Training

#### MI training

In total, patients needed 22.1 ± 9 (mean ± standard deviation) seconds for moving the 15 blocks mentally. A subdivision of the results into a first and a second half of the trials showed no significant difference (first half: 21.8 ± 8.4 s, second half: 22.3 ± 9.7 s; *p* = 0.35).

#### Hit

In total, patients needed 3.2 ± 1.1 s for deciding whether a right or a left hand was presented on the screen. The reaction time was significantly longer (*p* < 0.001) in the first run compared to the other runs (first run: 3.8 ± 1.3 s, second run: 3.1 ± 0.9 s, third run: 3.2 ± 0.8 s, fourth run: 2.9 ± 1 s). No significant difference was found between runs 2, 3 and 4.

Similarly, the number of correct responses was significantly lower in the first run compared to runs 3 (*p* = 0.006) and 4 (*p* = 0.002). There was no significant difference between runs 2, 3, and 4. Mean percentage of correct response: run 1, 80.4 ± 13.4%, run 2, 81.8 ± 20.9%, run 3, 86.6 ± 15.7%, run 4, 86.9 ± 16.4%.

### TMS results (Table 2)

TMS was performed in 26 patients. Seven patients had to be excluded due to presence of an exclusion criterion.

**Table 2:** Amplitudes of motor evoked potentials (MEP), measured in mV, and duration of the cortical silent period (cSP), measured in ms, before (pre) and after (post) a single session of mental chronometry training (MC) and hand-identification training (HIT) in *n* = 26 patients. Mean values ± standard deviations are presented. Aff, affected side; unaff, unaffected side

	MC MEP rest aff	MC MEP rest unaff	HIT MEP rest aff	HIT MEP rest unaff	MC MEP active aff	MC MEP active unaff	HIT MEP active aff	HIT MEP active unaff	MC cSP aff	MC cSP unaff	HIT cSP aff	HIT cSP unaff
Pre	0.53 ± 0.5	0.95 ± 0.6	0.48 ± 0.5	0.91 ± 0.8	4.0 ± 2.2	5.9 ± 2.5	4.5 ± 2.7	6.1 ± 2.6	208.0 ± 67.8	137.7 ± 39.2	224.4 ± 71.3	138.4 ± 42.2
post	0.65 ± 0.6	1.14 ± 0.6	0.62 ± 0.6	1.3 ± 1.1	4.2 ± 2.4	6.4 ± 3.3	4.4 ± 2.7	6.3 ± 2.4	200.4 ± 60.7	137.0 ± 46.6	214,8 ± 73.5	139.3 ± 45.1

MEP amplitudes at rest showed a significant difference between affected and unaffected side for MI training (*p* = 0.012) but not for HIT training (*p* = 0.052). No significant pre/post difference was found in either group.

MEP amplitudes during pre-innervation neither showed a significant difference between affected and unaffected side nor between pre and post values.

The cortical silent period showed significantly longer durations of the cSP in the affected side in both groups (MI training: p = 0.01; HIT training: p = 0.012). There were no significant differences between pre and post values.

## Discussion

The current study provides two main results: First, a single MI training modulated MC abilities and was associated with an improvement of motor execution. Second, MC changes were task-specific. Following MI training, stroke patients showed an improved MC performance whereas MC performance deteriorated after hand identification training. On the one hand, this result suggests that it is possible to improve MC abilities by a specific training. On the other hand, the observation suggests that focusing on another mental ability, in this case the performance of mental rotations, might interfere with MC performance, thus leading to an inferior MC ability.

Several studies have demonstrated that a single training session can induce a measurable change in performance (e.g., [28–32]). Our result is in line with this evidence.

However, task specificity has been investigated less consistently. In general, task-specific repetitive training has been shown to improve upper limb functions (e.g., [33, 34]). For motor imagery, a beneficial task-specific effect has also been described [35]. Our results provide further evidence for these findings. Notably, the exercises we used for comparison both address features of motor imagery. Thus, the control condition is more comparable to the main intervention than this has been the case in previous studies.

Interestingly, only after MI training a significant improvement of BBT motor execution was found. This finding suggests a task-specificity and indicates that even a single MI training may influence motor performance of the same task. In contrast, after HIT training, no significant improvement of the BBT motor execution was found. In future studies we will explore whether untrained motor tasks might also be enhanced after MI training.

Patients differed in their motor performance in relation to their sensory functions. Those without sensory deficits showed the best performance, those with severe sensory deficits the worst performance. This result replicates an earlier report [15]. We also replicated the finding that patients with the most severe sensory deficits had the worst MC ability prior to the training. However, the pre-post MC difference was similar in the three groups. Thus, it seems as if sensory deficits do not preclude the possibility to benefit from a motor imagery training. Nevertheless, this assumption needs to be verified in a larger group of patients with severe sensory impairments.

We were unable to demonstrate changes of motor excitability after the interventions. In contrast, it has been shown repeatedly that motor excitability is modulated during an motor imagery task [20, 36], thus suggesting that a single motor imagery training is not able to produce motor excitability changes that persist beyond the imagery session. In part, this may be due to a high variability of the data. However, the type of training we chose might also have an influence. A recent publication in which a single session of mirror therapy had been explored reported MEP increases in the affected limb after the training [37]. Apart from the different types of intervention, patient group characteristics might also contribute to this difference in MEP results.

In accordance with preceding studies, TMS results (MEP amplitudes and duration of the cSP) indicated significant differences between affected and unaffected side. (e.g., [38–40]).

Limitations of this study are that patients with severe motor deficits were not included and that it is not yet known if the improvements of motor functions are restricted to BBT performance or can be transferred to untrained motor tasks as well.

## Conclusions

The current study provides evidence that a single training session of mental chronometry can improve mental chronometry abilities and motor performance in a task-specific way. Based on this result, we hypothesize that a series of MC training sessions could strengthen the effect seen after a single session. Such a strengthened effect might then have the potential to further translate into improved motor execution.

## Data Availability

The datasets used and/or analysed during the current study are available from the corresponding author on reasonable request.

## Abbreviations

Aff.: Affected
ANOVA: Analysis of variance
BBT: Box and block test
cSP: Cortical silent period
EMG: Electromyography
FDI: First dorsal interosseous
HIT: Hand identification task
MC: Mental chronometry
ME: Motor execution
MEP: Motor evoked potential
MI: Motor imagery
ms: Milli seconds
mV: Milli volt
MT: Motor threshold
TMS: Transcranial magnetic stimulation
Unaff.: Unaffected
μV: Micro volt
